# *rac*-Dimethyl [(9-anthr­yl)(4-methyl­anilino)meth­yl]phospho­nate

**DOI:** 10.1107/S1600536811027711

**Published:** 2011-07-16

**Authors:** Ivanka Kraicheva, Ivelina Tsacheva, Elitsa Vodenicharova, Emil Tashev, Kolio Troev

**Affiliations:** a Institute of Polymers, Bulgarian Academy of Sciences, Acad. G. Bonchev str., bl. 103A, 1113 Sofia, Bulgaria

## Abstract

The title compound, C_24_H_24_NO_3_P, crystallizes as a racemate with two mol­ecules in the asymmetric unit. The structural features (bond lengths and angles) of the two mol­ecules are almost identical. The dihedral angle between the anthracene and toluidine rings is similar in the two mol­ecules, with values of 48.36 (9) and 51.15 (9)°. The methyl groups of one of the meth­oxy groups in one mol­ecule is disordered over two sets of sites, the major component having a site occupancy of 0.636 (3). In the crystal, both molecules are linked into inversion dimers by pairs of N—H⋯O hydrogen bonds.

## Related literature

For general background to the use of amino­phospho­nic acid derivatives in organic synthesis and as biologically active compounds, see: Cherkasov & Galkin (1998 ▶); Orsini *et al.* (2010 ▶); Green (2000 ▶); Rassukana *et al.* (2009 ▶); Kraicheva *et al.* (2011 ▶) and references therein.
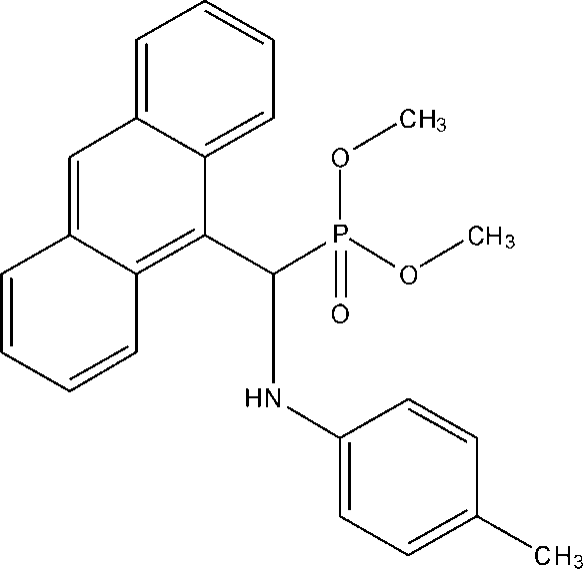


## Experimental

### 

#### Crystal data

C_24_H_24_NO_3_P
*M* *_r_* = 405.41
Triclinic, 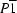

*a* = 7.944 (3) Å
*b* = 11.389 (4) Å
*c* = 24.007 (4) Å
α = 100.92 (4)°
β = 91.63 (3)°
γ = 95.17 (4)°
*V* = 2121.5 (11) Å^3^
*Z* = 4
Mo *K*α radiation
μ = 0.15 mm^−1^
*T* = 290 K
0.24 × 0.22 × 0.20 mm

#### Data collection

Enraf–Nonius CAD-4 diffractometer
8902 measured reflections
8275 independent reflections
2944 reflections with *I* > 2σ(*I*)
*R*_int_ = 0.085
3 standard reflections every 120 min  intensity decay: 1%

#### Refinement

*R*[*F* ^2^ > 2σ(*F* ^2^)] = 0.090
*wR*(*F* ^2^) = 0.278
*S* = 1.00
8275 reflections
539 parameters
H-atom parameters constrained
Δρ_max_ = 0.29 e Å^−3^
Δρ_min_ = −0.32 e Å^−3^

### 

Data collection: *CAD-4 EXPRESS* (Enraf–Nonius, 1994 ▶); cell refinement: *CAD-4 EXPRESS*; data reduction: *XCAD4* (Harms & Wocadlo, 1995) ▶; program(s) used to solve structure: *SHELXS97* (Sheldrick, 2008 ▶); program(s) used to refine structure: *SHELXL97* (Sheldrick, 2008 ▶); molecular graphics: *ORTEP-3 for Windows* (Farrugia, 1997 ▶); software used to prepare material for publication: *WinGX* (Farrugia, 1999 ▶).

## Supplementary Material

Crystal structure: contains datablock(s) I, global. DOI: 10.1107/S1600536811027711/kp2342sup1.cif

Structure factors: contains datablock(s) I. DOI: 10.1107/S1600536811027711/kp2342Isup2.hkl

Supplementary material file. DOI: 10.1107/S1600536811027711/kp2342Isup3.cml

Additional supplementary materials:  crystallographic information; 3D view; checkCIF report

## Figures and Tables

**Table 1:** Hydrogen-bond geometry (Å, °)

*D*—H⋯*A*	*D*—H	H⋯*A*	*D*⋯*A*	*D*—H⋯*A*
N1—H1⋯O11^i^	0.86	2.34	3.066 (6)	143
N2—H2⋯O21^ii^	0.86	2.62	3.239 (7)	130

Symmetry codes: (i) 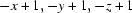; (ii) 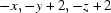.

**Fig. 1.**
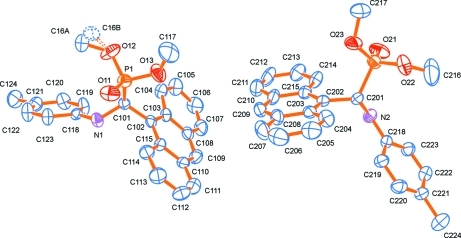
The asymmetric unit of title compound with the atom numbering scheme showing 50% probability displacement ellipsoids. H atoms have been omitted for clarity. The minor disorder component is shown as dasshed lines.

**Fig. 2.**
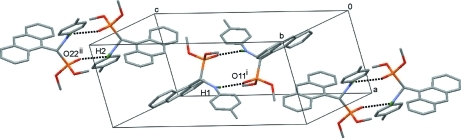
The packing arrangement of the molecules in the unit cell showing the hydrogen-bonding interactions as dashed lines [Symmetry codes: (i) 1 - x, 1 - y, 1 - z; (ii) -x, 2 - y, 2 - z].

## supplementary crystallographic information

### Comment

α-Aminophosphonic acid derivatives have gained widespread interest because of their versatile biological activity, that affords much opportunities of these compounds for pharmaceutical applications (Cherkasov & Galkin, 1998). They are considered to be bioisosteric phosphorus analogues of natural α-aminocarboxylic acids, in which the planar carboxylic acid group is replaced by a bulky phosphonic acid moiety (Orsini *et al.*, 2010). Due to the tetrahedral configuration at phosphorus, aminophosphonates serve as stable analogues of the unstable tetrahedral carbon intermediates formed in enzymatic processes and therefore act as enzyme inhibitors Rassukana *et al.* (2009). Numerous aminophosphonate derivatives are used as haptens for catalytic antibodies, metabolic regulators, antibiotics, as well as therapeutics, including antihypertensive, antibacterial, antiviral and antitumor agents. The title compound has been synthesised and tested for cytotoxicity on Balb/c 3 T3 (clone 31) cells, for *in vitro* antitumor activity using a panel of six human epithelial cancer cell lines and for genotoxicity and antiproliferative activity *in vivo* Kraicheva *et al.* (2011). Here we report its crystal structure.

The title compound (Fig. 1) possesses three distinct functional groups: anthracen, dimethyl phosphonate and *p*-toluidine. It crystallizes with two independent molecules in the assymetric unit. The anthracen and toluidine moieties are nearly planar (with respective r.m.s. of 0.076/0.008 and 0.065/0.009 Å for molecule A and B). The interplanar angle beteween the anthracen and the toluidine is 48.36 (9) and 51.15 (9)°, respectively. In the crystal structure of the studied compound symmetrically equivalent molecules of opposite chirality-enantiomers are connected by centrosymmetric N—H···O hydrogen bonds into dimmers (Fig. 2). One of the four methyl groups (form the two dimethyl phosphonate fragments) is disordered over two positions. The positional disorder on the C_methyl_ atom was resolved by finding alternative positions from the difference Fourier map, and was subsequently refined. The occupancy of the major component of the methyl group is 0.636 (3).

### Experimental

The studied compound was obtained according to Kraicheva *et al.* 2011. Suitable crystals were grown by slow evaporation from methanol/methylene chloride solution mixture (1:1 *v*/*v*) at room temperature.

### Refinement

All H atoms bonded to C or N were placed in idealised positions (C—H_aromatic_ = 0.93 Å, C—H_methine_ = 0.98 Å, *C*—H_methyl_ = 0.96 Å and N—H = 0.86 Å. All H atoms were constrained to ride on their parent atoms, with *U*_iso_(H) = 1.2*U*_eq_(C or N) and 1.5*U*_eq_(C_methyl_).

The positional disorder on the C methyl atom was resolved by finding alternative positions from the difference Fourier map, and was subsequently refined over two positions. The occupanciy of the major component of the methyl fragment is 0.636 (3).

### Crystal data

**Table:**

C_24_H_24_NO_3_P	*Z* = 4
*M**_r_* = 405.41	*F*(000) = 856
Triclinic, *P*1	*D*_x_ = 1.269 Mg m^−^^3^
Hall symbol: -P 1	Melting point = 452–453 K
*a* = 7.944 (3) Å	Mo *K*α radiation, λ = 0.71073 Å
*b* = 11.389 (4) Å	Cell parameters from 22 reflections
*c* = 24.007 (4) Å	θ = 16.0–17.8°
α = 100.92 (4)°	µ = 0.15 mm^−^^1^
β = 91.63 (3)°	*T* = 290 K
γ = 95.17 (4)°	Prism, colourless
*V* = 2121.5 (11) Å^3^	0.24 × 0.22 × 0.20 mm

### Data collection

**Table:**

Enraf–Nonius CAD-4 diffractometer	*R*_int_ = 0.085
Radiation source: Enraf Nonius FR590	θ_max_ = 26.0°, θ_min_ = 0.9°
graphite	*h* = 0→9
non–profiled ω/2τ scans	*k* = −14→13
8902 measured reflections	*l* = −29→29
8275 independent reflections	3 standard reflections every 120 min
2944 reflections with *I* > 2σ(*I*)	intensity decay: 1%

### Refinement

**Table:**

Refinement on *F*^2^	Primary atom site location: structure-invariant direct methods
Least-squares matrix: full	Secondary atom site location: difference Fourier map
*R*[*F*^2^ > 2σ(*F*^2^)] = 0.090	Hydrogen site location: inferred from neighbouring sites
*wR*(*F*^2^) = 0.278	H-atom parameters constrained
*S* = 1.00	*w* = 1/[σ^2^(*F*_o_^2^) + (0.0998*P*)^2^] where *P* = (*F*_o_^2^ + 2*F*_c_^2^)/3
8275 reflections	(Δ/σ)_max_ = 0.003
539 parameters	Δρ_max_ = 0.29 e Å^−^^3^
0 restraints	Δρ_min_ = −0.32 e Å^−^^3^

### Special details

**Table:**

Geometry. All e.s.d.'s (except the e.s.d. in the dihedral angle between two l.s. planes) are estimated using the full covariance matrix. The cell e.s.d.'s are taken into account individually in the estimation of e.s.d.'s in distances, angles and torsion angles; correlations between e.s.d.'s in cell parameters are only used when they are defined by crystal symmetry. An approximate (isotropic) treatment of cell e.s.d.'s is used for estimating e.s.d.'s involving l.s. planes.
Refinement. Refinement of *F*^2^ against ALL reflections. The weighted *R*-factor *wR* and goodness of fit *S* are based on *F*^2^, conventional *R*-factors *R* are based on *F*, with *F* set to zero for negative *F*^2^. The threshold expression of *F*^2^ > σ(*F*^2^) is used only for calculating *R*-factors(gt) *etc*. and is not relevant to the choice of reflections for refinement. *R*-factors based on *F*^2^ are statistically about twice as large as those based on *F*, and *R*- factors based on ALL data will be even larger.

### Fractional atomic coordinates and isotropic or equivalent isotropic displacement parameters (Å^2^)

**Table:**

	*x*	*y*	*z*	*U*_iso_*/*U*_eq_	Occ. (<1)
C101	0.5221 (8)	0.7612 (5)	0.5699 (2)	0.0376 (16)	
H101	0.5081	0.8407	0.5615	0.045*	
C102	0.5951 (7)	0.7858 (5)	0.6315 (3)	0.0388 (16)	
C103	0.6205 (8)	0.9055 (6)	0.6617 (3)	0.0436 (17)	
C104	0.5628 (9)	1.0076 (6)	0.6425 (3)	0.0520 (18)	
H104	0.5031	0.996	0.6077	0.062*	
C105	0.5929 (10)	1.1198 (6)	0.6735 (3)	0.070 (2)	
H105	0.5506	1.1834	0.6602	0.084*	
C106	0.6869 (11)	1.1428 (7)	0.7256 (4)	0.078 (3)	
H106	0.7104	1.2211	0.7456	0.094*	
C107	0.7426 (10)	1.0506 (7)	0.7462 (3)	0.069 (2)	
H107	0.8049	1.0663	0.7806	0.083*	
C108	0.7086 (8)	0.9296 (7)	0.7165 (3)	0.0500 (18)	
C109	0.7569 (9)	0.8358 (7)	0.7409 (3)	0.060 (2)	
H109	0.8158	0.8524	0.7759	0.073*	
C110	0.7177 (8)	0.7162 (7)	0.7130 (3)	0.0500 (18)	
C111	0.7586 (11)	0.6232 (8)	0.7402 (3)	0.075 (2)	
H111	0.8112	0.6418	0.7763	0.09*	
C112	0.7228 (11)	0.5074 (8)	0.7147 (4)	0.082 (3)	
H112	0.7511	0.4462	0.7329	0.098*	
C113	0.6424 (11)	0.4802 (7)	0.6604 (4)	0.071 (2)	
H113	0.6145	0.4001	0.6435	0.085*	
C114	0.6037 (9)	0.5671 (6)	0.6315 (3)	0.058 (2)	
H114	0.5562	0.5455	0.5947	0.069*	
C115	0.6357 (8)	0.6912 (6)	0.6574 (3)	0.0428 (16)	
C16A	0.217 (8)	0.722 (3)	0.4555 (8)	0.108 (13)	0.64 (7)
H16A	0.1501	0.7781	0.4424	0.163*	0.64 (7)
H16B	0.1661	0.6417	0.4418	0.163*	0.64 (7)
H16C	0.3291	0.7302	0.4417	0.163*	0.64 (7)
C16B	0.120 (7)	0.695 (4)	0.467 (3)	0.092 (15)	0.36 (7)
H16D	0.0777	0.7579	0.4504	0.138*	0.36 (7)
H16E	0.027	0.647	0.4784	0.138*	0.36 (7)
H16F	0.1808	0.6454	0.4389	0.138*	0.36 (7)
C117	0.0511 (11)	0.6710 (9)	0.6141 (4)	0.111 (3)	
H17A	−0.0063	0.6609	0.5776	0.167*	
H17B	0.0002	0.7298	0.6408	0.167*	
H17C	0.0422	0.5958	0.6268	0.167*	
C118	0.7049 (8)	0.7741 (5)	0.4881 (3)	0.0402 (16)	
C119	0.7440 (9)	0.8952 (6)	0.5003 (3)	0.060 (2)	
H119	0.7254	0.9374	0.5363	0.072*	
C120	0.8111 (10)	0.9574 (6)	0.4600 (3)	0.065 (2)	
H120	0.8342	1.0405	0.4694	0.078*	
C121	0.8434 (10)	0.8983 (7)	0.4070 (3)	0.061 (2)	
C122	0.8069 (10)	0.7770 (7)	0.3948 (3)	0.073 (2)	
H122	0.8303	0.7348	0.3592	0.087*	
C123	0.7351 (9)	0.7143 (6)	0.4344 (3)	0.0528 (19)	
H123	0.7075	0.6317	0.4244	0.063*	
C124	0.9191 (11)	0.9658 (7)	0.3632 (3)	0.084 (3)	
H24A	0.9343	1.0502	0.3789	0.127*	
H24B	0.8442	0.9517	0.33	0.127*	
H24C	1.0266	0.9378	0.353	0.127*	
C201	0.0364 (8)	0.7345 (5)	0.9271 (2)	0.0399 (16)	
H201	0.0158	0.6563	0.9383	0.048*	
C202	0.0977 (8)	0.7055 (6)	0.8668 (3)	0.0409 (16)	
C203	0.0967 (8)	0.5839 (6)	0.8392 (3)	0.0457 (17)	
C204	0.0218 (9)	0.4839 (6)	0.8613 (3)	0.060 (2)	
H204	−0.0307	0.4978	0.8957	0.072*	
C205	0.0264 (11)	0.3685 (7)	0.8325 (4)	0.077 (2)	
H205	−0.0241	0.3054	0.8477	0.092*	
C206	0.1059 (12)	0.3430 (9)	0.7805 (4)	0.087 (3)	
H206	0.1096	0.2639	0.7619	0.105*	
C207	0.1757 (11)	0.4331 (8)	0.7581 (3)	0.081 (3)	
H207	0.2293	0.4154	0.724	0.097*	
C208	0.1710 (9)	0.5562 (7)	0.7847 (3)	0.060 (2)	
C209	0.2349 (9)	0.6472 (8)	0.7585 (3)	0.062 (2)	
H209	0.2847	0.6284	0.7238	0.074*	
C210	0.2256 (9)	0.7655 (8)	0.7832 (3)	0.059 (2)	
C211	0.2790 (11)	0.8597 (9)	0.7548 (3)	0.076 (3)	
H211	0.3185	0.8396	0.7184	0.092*	
C212	0.2756 (12)	0.9752 (9)	0.7773 (4)	0.087 (3)	
H212	0.3172	1.0344	0.758	0.104*	
C213	0.2078 (10)	1.0066 (7)	0.8312 (3)	0.073 (2)	
H213	0.2019	1.0873	0.8466	0.087*	
C214	0.1508 (9)	0.9223 (6)	0.8612 (3)	0.059 (2)	
H214	0.1077	0.9462	0.8967	0.071*	
C215	0.1562 (8)	0.7976 (6)	0.8386 (3)	0.0460 (17)	
C216	−0.2316 (14)	0.7801 (11)	1.0400 (4)	0.139 (5)	
H16G	−0.1675	0.8573	1.046	0.208*	
H16H	−0.1735	0.7271	1.0588	0.208*	
H16I	−0.3413	0.7886	1.0553	0.208*	
C217	−0.4401 (10)	0.7696 (9)	0.8752 (4)	0.101 (3)	
H17D	−0.4973	0.7103	0.8453	0.151*	
H17E	−0.4406	0.8476	0.8656	0.151*	
H17F	−0.497	0.7687	0.9099	0.151*	
C218	0.2376 (8)	0.7492 (6)	1.0106 (3)	0.0442 (17)	
C219	0.2670 (8)	0.6297 (6)	1.0007 (3)	0.0491 (18)	
H219	0.2373	0.5819	0.9653	0.059*	
C220	0.3395 (9)	0.5800 (6)	1.0424 (3)	0.060 (2)	
H220	0.3562	0.4989	1.0344	0.072*	
C221	0.3879 (9)	0.6457 (7)	1.0953 (3)	0.059 (2)	
C222	0.3618 (10)	0.7680 (7)	1.1050 (3)	0.065 (2)	
H222	0.3934	0.8162	1.1402	0.079*	
C223	0.2902 (9)	0.8174 (6)	1.0631 (3)	0.0533 (19)	
H223	0.2767	0.8991	1.0703	0.064*	
C224	0.4689 (12)	0.5892 (8)	1.1404 (3)	0.094 (3)	
H24D	0.3828	0.5452	1.1576	0.141*	
H24E	0.5276	0.651	1.1689	0.141*	
H24F	0.5475	0.5355	1.1233	0.141*	
N1	0.6388 (7)	0.7089 (4)	0.5283 (2)	0.0470 (15)	
H1	0.6673	0.6379	0.5283	0.056*	
N2	0.1617 (7)	0.8036 (4)	0.9698 (2)	0.0470 (14)	
H2	0.1889	0.8784	0.9699	0.056*	
O11	0.3150 (6)	0.5491 (4)	0.5302 (2)	0.0685 (15)	
O12	0.2263 (8)	0.7445 (4)	0.5129 (2)	0.0842 (18)	
O13	0.2235 (6)	0.7100 (5)	0.6101 (2)	0.0815 (17)	
O21	−0.1452 (6)	0.9311 (4)	0.9540 (2)	0.0797 (17)	
O22	−0.2498 (7)	0.7329 (5)	0.9825 (2)	0.0760 (16)	
O23	−0.2654 (6)	0.7425 (5)	0.8827 (2)	0.0772 (16)	
P1	0.3154 (2)	0.67682 (16)	0.55329 (8)	0.0498 (6)	
P2	−0.1609 (2)	0.80141 (17)	0.93785 (8)	0.0507 (6)	

### Atomic displacement parameters (Å^2^)

**Table:**

	*U*^11^	*U*^22^	*U*^33^	*U*^12^	*U*^13^	*U*^23^
C101	0.044 (4)	0.032 (3)	0.039 (4)	0.008 (3)	0.007 (3)	0.009 (3)
C102	0.032 (4)	0.047 (4)	0.038 (4)	−0.001 (3)	0.011 (3)	0.010 (3)
C103	0.046 (4)	0.047 (4)	0.038 (4)	0.000 (3)	0.010 (3)	0.010 (3)
C104	0.066 (5)	0.043 (4)	0.044 (4)	−0.003 (4)	0.001 (4)	0.006 (3)
C105	0.094 (7)	0.047 (5)	0.064 (5)	−0.003 (4)	0.009 (5)	0.005 (4)
C106	0.090 (7)	0.052 (5)	0.082 (7)	0.003 (5)	−0.003 (5)	−0.011 (5)
C107	0.066 (6)	0.081 (6)	0.049 (5)	−0.001 (5)	−0.005 (4)	−0.015 (5)
C108	0.036 (4)	0.065 (5)	0.045 (4)	0.001 (4)	0.008 (3)	0.004 (4)
C109	0.050 (5)	0.082 (6)	0.048 (5)	0.000 (4)	−0.004 (4)	0.014 (4)
C110	0.040 (4)	0.068 (5)	0.050 (4)	0.018 (4)	0.009 (4)	0.024 (4)
C111	0.091 (7)	0.082 (6)	0.056 (5)	0.022 (5)	0.006 (5)	0.017 (5)
C112	0.097 (7)	0.083 (7)	0.078 (6)	0.037 (6)	0.000 (5)	0.039 (5)
C113	0.091 (7)	0.054 (5)	0.076 (6)	0.018 (4)	0.008 (5)	0.025 (4)
C114	0.068 (5)	0.051 (5)	0.059 (5)	0.013 (4)	0.008 (4)	0.021 (4)
C115	0.045 (4)	0.048 (4)	0.038 (4)	0.006 (3)	0.004 (3)	0.013 (3)
C16A	0.11 (3)	0.18 (2)	0.050 (11)	0.06 (2)	0.016 (13)	0.020 (11)
C16B	0.03 (2)	0.17 (3)	0.07 (3)	0.02 (2)	−0.011 (17)	−0.01 (2)
C117	0.058 (7)	0.151 (9)	0.121 (9)	−0.012 (6)	0.021 (6)	0.026 (7)
C118	0.052 (4)	0.033 (4)	0.036 (4)	0.008 (3)	−0.004 (3)	0.006 (3)
C119	0.078 (6)	0.053 (5)	0.047 (4)	0.001 (4)	0.028 (4)	0.005 (4)
C120	0.078 (6)	0.046 (4)	0.071 (6)	0.001 (4)	0.021 (5)	0.012 (4)
C121	0.071 (6)	0.067 (5)	0.052 (5)	0.012 (4)	0.010 (4)	0.023 (4)
C122	0.094 (7)	0.073 (6)	0.047 (5)	0.002 (5)	0.019 (5)	0.000 (4)
C123	0.067 (5)	0.047 (4)	0.040 (4)	−0.007 (4)	0.012 (4)	0.001 (3)
C124	0.098 (7)	0.091 (6)	0.075 (6)	0.007 (5)	0.026 (5)	0.041 (5)
C201	0.044 (4)	0.041 (4)	0.036 (4)	0.006 (3)	−0.003 (3)	0.012 (3)
C202	0.032 (4)	0.052 (4)	0.040 (4)	0.006 (3)	−0.001 (3)	0.011 (3)
C203	0.036 (4)	0.055 (5)	0.042 (4)	0.006 (3)	−0.006 (3)	0.000 (3)
C204	0.062 (5)	0.052 (5)	0.060 (5)	−0.002 (4)	0.004 (4)	−0.001 (4)
C205	0.073 (6)	0.065 (6)	0.086 (7)	0.003 (5)	−0.012 (5)	0.002 (5)
C206	0.076 (7)	0.078 (7)	0.091 (8)	0.017 (6)	−0.017 (6)	−0.026 (6)
C207	0.076 (7)	0.092 (7)	0.059 (6)	0.013 (6)	0.008 (5)	−0.026 (5)
C208	0.056 (5)	0.079 (6)	0.040 (4)	0.009 (4)	0.001 (4)	−0.004 (4)
C209	0.042 (5)	0.099 (7)	0.042 (4)	0.011 (4)	0.005 (4)	0.002 (5)
C210	0.054 (5)	0.088 (6)	0.036 (4)	−0.003 (4)	0.001 (4)	0.018 (4)
C211	0.072 (6)	0.106 (7)	0.052 (5)	0.007 (6)	0.009 (4)	0.016 (5)
C212	0.102 (8)	0.111 (8)	0.055 (6)	−0.018 (6)	−0.002 (5)	0.050 (6)
C213	0.088 (7)	0.067 (5)	0.064 (6)	−0.007 (5)	−0.004 (5)	0.024 (5)
C214	0.077 (6)	0.059 (5)	0.043 (4)	−0.010 (4)	−0.003 (4)	0.019 (4)
C215	0.039 (4)	0.054 (5)	0.043 (4)	−0.003 (3)	−0.004 (3)	0.008 (3)
C216	0.128 (10)	0.198 (13)	0.083 (8)	−0.021 (9)	0.039 (7)	0.024 (8)
C217	0.060 (6)	0.146 (9)	0.096 (7)	0.028 (6)	−0.014 (5)	0.018 (6)
C218	0.042 (4)	0.057 (5)	0.035 (4)	0.007 (3)	0.003 (3)	0.010 (3)
C219	0.060 (5)	0.041 (4)	0.044 (4)	0.014 (3)	−0.008 (4)	−0.001 (3)
C220	0.064 (5)	0.051 (5)	0.068 (5)	0.014 (4)	0.001 (4)	0.019 (4)
C221	0.049 (5)	0.084 (6)	0.050 (5)	0.019 (4)	0.001 (4)	0.026 (4)
C222	0.073 (6)	0.083 (6)	0.045 (5)	0.017 (5)	0.004 (4)	0.018 (4)
C223	0.062 (5)	0.052 (4)	0.044 (4)	0.013 (4)	−0.003 (4)	0.003 (4)
C224	0.103 (8)	0.118 (8)	0.072 (6)	0.023 (6)	−0.008 (5)	0.042 (6)
N1	0.067 (4)	0.031 (3)	0.047 (3)	0.012 (3)	0.017 (3)	0.010 (3)
N2	0.052 (4)	0.044 (3)	0.046 (3)	−0.001 (3)	−0.002 (3)	0.014 (3)
O11	0.061 (4)	0.039 (3)	0.100 (4)	0.002 (2)	−0.011 (3)	0.001 (3)
O12	0.107 (5)	0.066 (4)	0.076 (4)	0.014 (3)	−0.046 (4)	0.012 (3)
O13	0.044 (3)	0.117 (5)	0.071 (4)	−0.006 (3)	0.015 (3)	−0.010 (3)
O21	0.064 (4)	0.054 (3)	0.121 (5)	0.020 (3)	0.010 (3)	0.010 (3)
O22	0.079 (4)	0.078 (4)	0.068 (4)	−0.001 (3)	0.032 (3)	0.007 (3)
O23	0.044 (3)	0.113 (4)	0.069 (4)	0.020 (3)	−0.012 (3)	0.000 (3)
P1	0.0489 (12)	0.0455 (11)	0.0529 (12)	0.0063 (9)	−0.0024 (10)	0.0041 (9)
P2	0.0466 (13)	0.0537 (13)	0.0515 (12)	0.0109 (10)	0.0074 (10)	0.0058 (10)

### Geometric parameters (Å, °)

**Table:**

C101—N1	1.463 (7)	C201—P2	1.808 (6)
C101—C102	1.538 (8)	C201—H201	0.98
C101—P1	1.820 (6)	C202—C215	1.404 (8)
C101—H101	0.98	C202—C203	1.416 (8)
C102—C115	1.398 (8)	C203—C204	1.434 (9)
C102—C103	1.412 (8)	C203—C208	1.442 (9)
C103—C104	1.435 (8)	C204—C205	1.369 (9)
C103—C108	1.440 (9)	C204—H204	0.93
C104—C105	1.349 (9)	C205—C206	1.409 (12)
C104—H104	0.93	C205—H205	0.93
C105—C106	1.405 (10)	C206—C207	1.331 (11)
C105—H105	0.93	C206—H206	0.93
C106—C107	1.345 (10)	C207—C208	1.431 (10)
C106—H106	0.93	C207—H207	0.93
C107—C108	1.425 (9)	C208—C209	1.377 (10)
C107—H107	0.93	C209—C210	1.375 (10)
C108—C109	1.389 (9)	C209—H209	0.93
C109—C110	1.402 (9)	C210—C211	1.418 (10)
C109—H109	0.93	C210—C215	1.449 (9)
C110—C111	1.401 (9)	C211—C212	1.327 (11)
C110—C115	1.437 (9)	C211—H211	0.93
C111—C112	1.346 (10)	C212—C213	1.410 (11)
C111—H111	0.93	C212—H212	0.93
C112—C113	1.403 (10)	C213—C214	1.359 (9)
C112—H112	0.93	C213—H213	0.93
C113—C114	1.364 (9)	C214—C215	1.426 (9)
C113—H113	0.93	C214—H214	0.93
C114—C115	1.429 (9)	C216—O22	1.383 (10)
C114—H114	0.93	C216—H16G	0.96
C16A—O12	1.350 (18)	C216—H16H	0.96
C16A—H16A	0.96	C216—H16I	0.96
C16A—H16B	0.96	C217—O23	1.462 (9)
C16A—H16C	0.96	C217—H17D	0.96
C16B—O12	1.37 (3)	C217—H17E	0.96
C16B—H16D	0.96	C217—H17F	0.96
C16B—H16E	0.96	C218—C219	1.379 (8)
C16B—H16F	0.96	C218—C223	1.381 (8)
C117—O13	1.413 (9)	C218—N2	1.402 (7)
C117—H17A	0.96	C219—C220	1.377 (9)
C117—H17B	0.96	C219—H219	0.93
C117—H17C	0.96	C220—C221	1.370 (9)
C118—C119	1.360 (8)	C220—H220	0.93
C118—C123	1.378 (8)	C221—C222	1.403 (10)
C118—N1	1.409 (8)	C221—C224	1.517 (9)
C119—C120	1.393 (9)	C222—C223	1.375 (9)
C119—H119	0.93	C222—H222	0.93
C120—C121	1.366 (10)	C223—H223	0.93
C120—H120	0.93	C224—H24D	0.96
C121—C122	1.359 (10)	C224—H24E	0.96
C121—C124	1.522 (9)	C224—H24F	0.96
C122—C123	1.398 (10)	N1—H1	0.86
C122—H122	0.93	N2—H2	0.86
C123—H123	0.93	O11—P1	1.455 (5)
C124—H24A	0.96	O12—P1	1.542 (5)
C124—H24B	0.96	O13—P1	1.563 (5)
C124—H24C	0.96	O21—P2	1.447 (5)
C201—N2	1.469 (7)	O22—P2	1.586 (5)
C201—C202	1.526 (8)	O23—P2	1.546 (5)
			
N1—C101—C102	113.6 (5)	C202—C203—C204	124.2 (6)
N1—C101—P1	108.0 (4)	C202—C203—C208	119.3 (7)
C102—C101—P1	118.9 (4)	C204—C203—C208	116.5 (6)
N1—C101—H101	105	C205—C204—C203	121.1 (7)
C102—C101—H101	105	C205—C204—H204	119.4
P1—C101—H101	105	C203—C204—H204	119.4
C115—C102—C103	120.3 (6)	C204—C205—C206	121.5 (9)
C115—C102—C101	120.6 (5)	C204—C205—H205	119.3
C103—C102—C101	119.0 (5)	C206—C205—H205	119.3
C102—C103—C104	125.1 (6)	C207—C206—C205	119.5 (8)
C102—C103—C108	118.8 (6)	C207—C206—H206	120.3
C104—C103—C108	116.1 (6)	C205—C206—H206	120.3
C105—C104—C103	121.8 (7)	C206—C207—C208	122.2 (8)
C105—C104—H104	119.1	C206—C207—H207	118.9
C103—C104—H104	119.1	C208—C207—H207	118.9
C104—C105—C106	121.4 (8)	C209—C208—C207	120.5 (7)
C104—C105—H105	119.3	C209—C208—C203	120.4 (7)
C106—C105—H105	119.3	C207—C208—C203	119.1 (8)
C107—C106—C105	119.5 (7)	C210—C209—C208	120.6 (7)
C107—C106—H106	120.2	C210—C209—H209	119.7
C105—C106—H106	120.2	C208—C209—H209	119.7
C106—C107—C108	121.6 (7)	C209—C210—C211	121.2 (8)
C106—C107—H107	119.2	C209—C210—C215	120.9 (7)
C108—C107—H107	119.2	C211—C210—C215	117.9 (7)
C109—C108—C107	120.2 (7)	C212—C211—C210	123.4 (8)
C109—C108—C103	120.4 (7)	C212—C211—H211	118.3
C107—C108—C103	119.3 (7)	C210—C211—H211	118.3
C108—C109—C110	120.6 (7)	C211—C212—C213	118.7 (8)
C108—C109—H109	119.7	C211—C212—H212	120.6
C110—C109—H109	119.7	C213—C212—H212	120.6
C111—C110—C109	119.4 (7)	C214—C213—C212	122.0 (8)
C111—C110—C115	121.2 (7)	C214—C213—H213	119
C109—C110—C115	119.4 (6)	C212—C213—H213	119
C112—C111—C110	120.9 (8)	C213—C214—C215	120.6 (7)
C112—C111—H111	119.5	C213—C214—H214	119.7
C110—C111—H111	119.5	C215—C214—H214	119.7
C111—C112—C113	119.1 (7)	C202—C215—C214	123.8 (6)
C111—C112—H112	120.4	C202—C215—C210	118.8 (6)
C113—C112—H112	120.4	C214—C215—C210	117.4 (7)
C114—C113—C112	122.4 (8)	O22—C216—H16G	109.5
C114—C113—H113	118.8	O22—C216—H16H	109.5
C112—C113—H113	118.8	H16G—C216—H16H	109.5
C113—C114—C115	120.4 (7)	O22—C216—H16I	109.5
C113—C114—H114	119.8	H16G—C216—H16I	109.5
C115—C114—H114	119.8	H16H—C216—H16I	109.5
C102—C115—C114	124.1 (6)	O23—C217—H17D	109.5
C102—C115—C110	120.0 (6)	O23—C217—H17E	109.5
C114—C115—C110	115.9 (6)	H17D—C217—H17E	109.5
O12—C16A—H16A	109.5	O23—C217—H17F	109.5
O12—C16A—H16B	109.5	H17D—C217—H17F	109.5
H16A—C16A—H16B	109.5	H17E—C217—H17F	109.5
O12—C16A—H16C	109.5	C219—C218—C223	117.1 (6)
H16A—C16A—H16C	109.5	C219—C218—N2	123.2 (6)
H16B—C16A—H16C	109.5	C223—C218—N2	119.6 (6)
O12—C16B—H16D	109.5	C220—C219—C218	121.1 (6)
O12—C16B—H16E	109.5	C220—C219—H219	119.5
H16D—C16B—H16E	109.5	C218—C219—H219	119.5
O12—C16B—H16F	109.5	C221—C220—C219	122.4 (7)
H16D—C16B—H16F	109.5	C221—C220—H220	118.8
H16E—C16B—H16F	109.5	C219—C220—H220	118.8
O13—C117—H17A	109.5	C220—C221—C222	116.6 (6)
O13—C117—H17B	109.5	C220—C221—C224	121.5 (7)
H17A—C117—H17B	109.5	C222—C221—C224	121.9 (7)
O13—C117—H17C	109.5	C223—C222—C221	120.8 (7)
H17A—C117—H17C	109.5	C223—C222—H222	119.6
H17B—C117—H17C	109.5	C221—C222—H222	119.6
C119—C118—C123	117.6 (6)	C222—C223—C218	121.9 (7)
C119—C118—N1	122.6 (6)	C222—C223—H223	119
C123—C118—N1	119.8 (6)	C218—C223—H223	119
C118—C119—C120	121.6 (6)	C221—C224—H24D	109.5
C118—C119—H119	119.2	C221—C224—H24E	109.5
C120—C119—H119	119.2	H24D—C224—H24E	109.5
C121—C120—C119	121.0 (7)	C221—C224—H24F	109.5
C121—C120—H120	119.5	H24D—C224—H24F	109.5
C119—C120—H120	119.5	H24E—C224—H24F	109.5
C122—C121—C120	117.7 (7)	C118—N1—C101	120.5 (5)
C122—C121—C124	121.1 (7)	C118—N1—H1	119.7
C120—C121—C124	121.2 (7)	C101—N1—H1	119.7
C121—C122—C123	121.6 (7)	C218—N2—C201	121.0 (5)
C121—C122—H122	119.2	C218—N2—H2	119.5
C123—C122—H122	119.2	C201—N2—H2	119.5
C118—C123—C122	120.4 (7)	C16A—O12—P1	128.7 (11)
C118—C123—H123	119.8	C16B—O12—P1	127 (2)
C122—C123—H123	119.8	C117—O13—P1	121.2 (5)
C121—C124—H24A	109.5	C216—O22—P2	120.8 (6)
C121—C124—H24B	109.5	C217—O23—P2	119.7 (5)
H24A—C124—H24B	109.5	O11—P1—O12	112.9 (3)
C121—C124—H24C	109.5	O11—P1—O13	115.9 (3)
H24A—C124—H24C	109.5	O12—P1—O13	103.5 (3)
H24B—C124—H24C	109.5	O11—P1—C101	116.3 (3)
N2—C201—C202	115.3 (5)	O12—P1—C101	104.2 (3)
N2—C201—P2	107.6 (4)	O13—P1—C101	102.5 (3)
C202—C201—P2	118.4 (4)	O21—P2—O23	118.1 (3)
N2—C201—H201	104.7	O21—P2—O22	114.8 (3)
C202—C201—H201	104.7	O23—P2—O22	101.1 (3)
P2—C201—H201	104.7	O21—P2—C201	115.3 (3)
C215—C202—C203	119.7 (6)	O23—P2—C201	102.0 (3)
C215—C202—C201	120.9 (6)	O22—P2—C201	103.4 (3)
C203—C202—C201	119.4 (6)		
			
N1—C101—C102—C115	−64.1 (7)	C204—C203—C208—C207	−4.0 (10)
P1—C101—C102—C115	64.7 (7)	C207—C208—C209—C210	177.9 (7)
N1—C101—C102—C103	116.0 (6)	C203—C208—C209—C210	−1.4 (11)
P1—C101—C102—C103	−115.2 (6)	C208—C209—C210—C211	−175.1 (7)
C115—C102—C103—C104	−171.1 (6)	C208—C209—C210—C215	3.0 (11)
C101—C102—C103—C104	8.8 (9)	C209—C210—C211—C212	−178.6 (8)
C115—C102—C103—C108	8.0 (9)	C215—C210—C211—C212	3.1 (12)
C101—C102—C103—C108	−172.1 (6)	C210—C211—C212—C213	−3.3 (14)
C102—C103—C104—C105	−179.5 (7)	C211—C212—C213—C214	2.0 (13)
C108—C103—C104—C105	1.4 (10)	C212—C213—C214—C215	−0.6 (12)
C103—C104—C105—C106	1.9 (12)	C203—C202—C215—C214	175.3 (6)
C104—C105—C106—C107	−2.7 (13)	C201—C202—C215—C214	−5.2 (10)
C105—C106—C107—C108	−0.1 (13)	C203—C202—C215—C210	−4.4 (9)
C106—C107—C108—C109	−175.2 (8)	C201—C202—C215—C210	175.2 (6)
C106—C107—C108—C103	3.5 (11)	C213—C214—C215—C202	−179.2 (7)
C102—C103—C108—C109	−4.5 (10)	C213—C214—C215—C210	0.5 (10)
C104—C103—C108—C109	174.7 (6)	C209—C210—C215—C202	−0.2 (10)
C102—C103—C108—C107	176.8 (6)	C211—C210—C215—C202	178.1 (6)
C104—C103—C108—C107	−4.0 (9)	C209—C210—C215—C214	−179.8 (7)
C107—C108—C109—C110	177.3 (7)	C211—C210—C215—C214	−1.6 (10)
C103—C108—C109—C110	−1.3 (10)	C223—C218—C219—C220	2.5 (10)
C108—C109—C110—C111	−176.0 (7)	N2—C218—C219—C220	−178.9 (6)
C108—C109—C110—C115	3.7 (10)	C218—C219—C220—C221	−0.8 (11)
C109—C110—C111—C112	−180.0 (8)	C219—C220—C221—C222	−0.7 (11)
C115—C110—C111—C112	0.3 (12)	C219—C220—C221—C224	−179.5 (7)
C110—C111—C112—C113	−0.4 (13)	C220—C221—C222—C223	0.4 (11)
C111—C112—C113—C114	2.2 (13)	C224—C221—C222—C223	179.2 (7)
C112—C113—C114—C115	−3.7 (12)	C221—C222—C223—C218	1.5 (12)
C103—C102—C115—C114	175.6 (6)	C219—C218—C223—C222	−2.9 (11)
C101—C102—C115—C114	−4.3 (10)	N2—C218—C223—C222	178.5 (7)
C103—C102—C115—C110	−5.7 (9)	C119—C118—N1—C101	35.5 (9)
C101—C102—C115—C110	174.4 (6)	C123—C118—N1—C101	−145.7 (6)
C113—C114—C115—C102	−177.9 (7)	C102—C101—N1—C118	−113.7 (6)
C113—C114—C115—C110	3.3 (10)	P1—C101—N1—C118	112.1 (5)
C111—C110—C115—C102	179.5 (7)	C219—C218—N2—C201	30.8 (9)
C109—C110—C115—C102	−0.2 (10)	C223—C218—N2—C201	−150.7 (6)
C111—C110—C115—C114	−1.7 (10)	C202—C201—N2—C218	−107.5 (6)
C109—C110—C115—C114	178.6 (6)	P2—C201—N2—C218	117.9 (5)
C123—C118—C119—C120	0.4 (11)	C16A—O12—P1—O11	−32 (4)
N1—C118—C119—C120	179.2 (7)	C16B—O12—P1—O11	16 (4)
C118—C119—C120—C121	−1.4 (12)	C16A—O12—P1—O13	−158 (4)
C119—C120—C121—C122	0.4 (12)	C16B—O12—P1—O13	−110 (4)
C119—C120—C121—C124	−179.1 (7)	C16A—O12—P1—C101	95 (4)
C120—C121—C122—C123	1.5 (12)	C16B—O12—P1—C101	143 (4)
C124—C121—C122—C123	−179.1 (7)	C117—O13—P1—O11	−57.8 (7)
C119—C118—C123—C122	1.4 (11)	C117—O13—P1—O12	66.3 (7)
N1—C118—C123—C122	−177.4 (7)	C117—O13—P1—C101	174.4 (7)
C121—C122—C123—C118	−2.4 (12)	N1—C101—P1—O11	34.0 (5)
N2—C201—C202—C215	−59.1 (8)	C102—C101—P1—O11	−97.4 (5)
P2—C201—C202—C215	70.3 (7)	N1—C101—P1—O12	−90.9 (4)
N2—C201—C202—C203	120.4 (6)	C102—C101—P1—O12	137.7 (5)
P2—C201—C202—C203	−110.2 (6)	N1—C101—P1—O13	161.5 (4)
C215—C202—C203—C204	−172.4 (6)	C102—C101—P1—O13	30.1 (5)
C201—C202—C203—C204	8.1 (9)	C217—O23—P2—O21	−54.9 (7)
C215—C202—C203—C208	6.0 (9)	C217—O23—P2—O22	71.2 (6)
C201—C202—C203—C208	−173.5 (6)	C217—O23—P2—C201	177.6 (6)
C202—C203—C204—C205	−179.5 (7)	C216—O22—P2—O21	−32.4 (8)
C208—C203—C204—C205	2.0 (10)	C216—O22—P2—O23	−160.7 (7)
C203—C204—C205—C206	0.6 (12)	C216—O22—P2—C201	94.0 (8)
C204—C205—C206—C207	−1.2 (13)	N2—C201—P2—O21	37.4 (5)
C205—C206—C207—C208	−0.9 (14)	C202—C201—P2—O21	−95.6 (5)
C206—C207—C208—C209	−175.8 (8)	N2—C201—P2—O23	166.7 (4)
C206—C207—C208—C203	3.6 (12)	C202—C201—P2—O23	33.7 (6)
C202—C203—C208—C209	−3.2 (10)	N2—C201—P2—O22	−88.7 (4)
C204—C203—C208—C209	175.4 (6)	C202—C201—P2—O22	138.4 (5)
C202—C203—C208—C207	177.5 (6)		

### Hydrogen-bond geometry (Å, °)

**Table:**

*D*—H···*A*	*D*—H	H···*A*	*D*···*A*	*D*—H···*A*
N1—H1···O11^i^	0.86	2.34	3.066 (6)	143.
N2—H2···O21^ii^	0.86	2.62	3.239 (7)	130.

Symmetry codes: (i) −*x*+1, −*y*+1, −*z*+1; (ii) −*x*, −*y*+2, −*z*+2.
